# The T cell receptor resides in ordered plasma membrane nanodomains that aggregate upon patching of the receptor

**DOI:** 10.1038/srep10082

**Published:** 2015-05-08

**Authors:** Jelena Dinic, Astrid Riehl, Jeremy Adler, Ingela Parmryd

**Affiliations:** 1 Science for Life Laboratory, Department of Medical Cell Biology, Uppsala University, 751 23 Uppsala, Sweden

## Abstract

Two related models for T cell signalling initiation suggest either that T cell receptor (TCR) engagement leads to its recruitment to ordered membrane domains, often referred to as lipid rafts, where signalling molecules are enriched or that ordered TCR-containing membrane nanodomains coalesce upon TCR engagement. That ordered domains form upon TCR engagement, as they do upon lipid raft marker patching, has not been considered. The target of this study was to differentiate between those three options. Plasma membrane order was followed in live T cells at 37 °C using laurdan to report on lipid packing. Patching of the TCR that elicits a signalling response resulted in aggregation, not formation, of ordered plasma membrane domains in both Jurkat and primary T cells. The TCR colocalised with actin filaments at the plasma membrane in unstimulated Jurkat T cells, consistent with it being localised to ordered membrane domains. The colocalisation was most prominent in cells in G1 phase when the cells are ready to commit to proliferation. At other cell cycle phases the TCR was mainly found at perinuclear membranes. Our study suggests that the TCR resides in ordered plasma membrane domains that are linked to actin filaments and aggregate upon TCR engagement.

Ordered membrane nanodomains, often referred to as lipid rafts, are implicated in immune cell signalling. They are considered to form by the self-aggregation of cholesterol and sphingolipids^1^ and are believed to exist as liquid ordered (lo) domains, in contrast to the rest of the membrane that exists liquid disordered (ld) domains. However, both are liquid phases and diffusion can take place inside, around as well as into and out of the domains so membrane components continuously shift between domains and their surroundings.

T cell signalling is initiated by Src family tyrosine kinases, Lck and Fyn, by phosphorylation of immunoreceptor tyrosine based activation motifs (ITAMs) in the CD3 subunits of the T cell receptor (TCR). Downstream signalling involves the activation of Ras and calcium pathways. All these pathways can be activated by crosslinking different lipid raft components, the ganglioside GM1 or the GPI-anchored protein CD59, suggesting a link between the aggregation of ordered membrane nanodomains and early T cell signalling^2^,^3^,^4^. Cold stress and moderate cholesterol depletion can also lead to lipid raft aggregation and T cell activation^5^,^6^.

Formation of an immunological synapse (IS) occurs after the initial signalling events^7^ and is accomplished by the transport of microclusters containing the TCR and signalling proteins along both actin filaments and microtubules to form a central supramolecular activation cluster (cSMAC)^8^,^9^,^10^. The IS in both fixed and live T cells has been shown to contain ordered membrane domains^11^,^12^. In addition the IS in fixed cells is enriched in lo-domain partitioning signalling molecules^13^. However, the lipid packing in the TCR-containing microclusters has not been studied.

Using total internal fluorescence microscopy (TIRF) it has recently been suggested that TCR microclusters exist in resting T cells^14^ although TCR microclusters are not generally observed in resting T cells using other fluorescence microscopy methods. Whether or not the TCR exists exclusively as monomers or a mix of monomers and dimer/multimer clusters also seems to reflect the choice of methodology^15^,^16^. However, super resolution studies suggest that the TCR in resting T cells resides in nanodomains^17^ which is not incompatible with the TCR existing as monomers since within the nanodomains there could be lipids that preclude direct interaction of individual TCRs but still lead to a TCR density required to respond to scarce agonists^15^.

Remodelling of the actin cytoskeleton is integral to T cell activation^18^,^19^. Polymerised actin has long been known to accumulate at capping sites of the TCRs^20^. Polymerised actin also accumulates underneath aggregated lipid rafts^21^. Moreover, ordered lipid domains form at attachment points between actin filaments and the plasma membrane in a phosphoinositide dependent manner, further strengthening the link between lipid rafts and the cytoskeleton^22^.

In this study, we have used the probe laurdan to assess the plasma membrane order in live Jurkat and primary human T cells upon initiation of signalling by antibodies directed at the TCR subunit CD3 in order to address the nature of the lipid environment in TCR nano- and microdomains. Our data provides answers to the questions of whether the TCR is a lipid raft resident protein or is recruited to lipid rafts upon T cell activation and whether lipid rafts form upon aggregation of the TCR.

## Results

There is consensus that the TCR is found in ordered plasma membrane domains after its engagement/the formation of the immunological synapse but there are three possible scenarios compatible with this notion. The first is that the TCR is recruited to ordered plasma membrane nanodomains upon its engagement, the second that the TCR always resides in ordered plasma membrane domains and the third that ordered plasma membrane domains form upon engagement of the TCR.

### The TCR resides in small ordered domains in the plasma membrane of resting T cells

**Figure 1:**
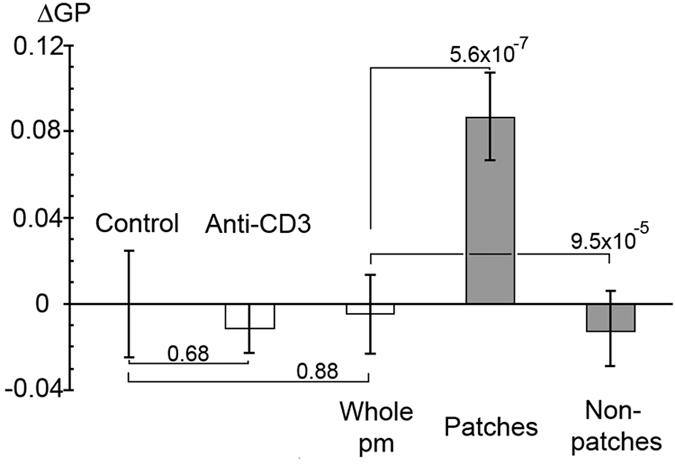
The TCR resides in ordered plasma membrane domains prior to Jurkat T cell stimulation. Cells were patched by incubation on ice with anti-CD3 (OKT3) for 30 min and anti-mouse Alexa Fluor-647 for 30 min. Cells were imaged after 20–25 min at 37 °C. The GP values for the control cells were normalized to 0. Data shown are mean ± s. e. m.. p values are from a two-tailed t-test with the control and from a paired two-tailed t-test with the whole plasma membrane of patched cells, n=28-30.

**Figure 2:**
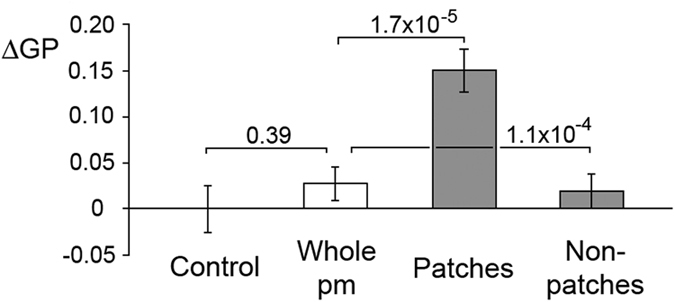
The TCR resides in ordered plasma membrane domains in resting human primary T cells. Primary T cells were patched by incubation on ice with anti-CD3 (OKT3) for 30 min and anti-mouse Alexa Fluor-647 for 30 min. Cells were imaged after 20–25 min at 37 °C. Data shown are mean ± s. e. m.. p values are from a two-tailed t-test with the control and from a paired two-tailed t-test with the whole plasma membrane of patched cells, n=27-30

To address the outstanding questions whether TCR nanoclusters in resting cells have the expected features of lipid rafts and whether ordered membrane domains form upon TCR ligation, laurdan labelled T cells were imaged live and the generalised polarisation (GP) values, a measure of the relative proportion of lo and ld phase in the membrane^23^, calculated. Since the aggregation of GM1 and CD59 leads to the formation of ordered plasma membrane domains^22^, it seemed possible that TCR patching also could lead to their formation. The TCR was patched using a monoclonal antibody against its CD3ε subunit. Anti-CD3ε treatment alone, which is sufficient to trigger a signalling response, did not cause any change in the relative proportion of disordered and ordered domains in Jurkat T cells (Fig. 1). Nor did patching of CD3ε with a secondary antibody, which causes TCR patching. However, the patching caused an enrichment of ordered domains in the TCR patches and a corresponding depletion of ordered domains in the non-patched plasma membrane regions. The patched regions occupied only about 7% of the entire plasma membrane, explaining why the increase of ordered domains in the patched areas is greater than their decrease in non-patched areas. The result is consistent with small ordered domains aggregating upon TCR triggering but not with the *de novo* formation of ordered domains upon TCR engagement. Similar results were obtained upon TCR-patching of human primary T cells (Fig. 2), strengthening the conclusion that the TCR is found in ordered membrane domains in resting T cells.

During the TCR crosslinking the cells are subjected to several large temperature changes (from 37 °C to 0 °C and back to 37 °C). To assess whether this influenced the relative proportion of lo and ld domains in the plasma membrane we compared the GP values between cell populations continuously kept at 37 °C to those that had recoved for 15 min at 37 °C after a 30 min incubation on ice. The relative proportion of lo and ld domains in the plasma membrane did not significantly differ between these two populations (Fig. S1). We therefore conclude that any membrane phase alterations caused by temperature changes are temporary and would not be reflected in our experiments.

### Aggregation of TCR nanodomains does not lead to an increase in plasma membrane attached actin filaments

We have shown that ordered plasma membrane domains form when actin filaments are attached to the plasma membrane in a phosphoinositide-dependent manner^22^. To address actin filament dynamics upon TCR patching, the cells were analysed for their cell peripheral actin staining intensity. Jurkat T cells were patched for TCR and fixed. Cells were labelled with FITC-phalloidin, a toxin that binds to actin filaments, and DiI-C12, to identify the plasma membrane, and imaged by confocal microscopy. FITC-phalloidin staining was measured exclusively at the cell periphery. Consistent with TCR-patching not causing the formation of ordered membrane domains, TCR-patching did not cause an increase in the amount of cell peripheral actin staining (Table 1). However, under the TCR-patches the FITC phalloidin staining was more intense than in the rest of the plasma membrane. This suggests that the TCR in resting T cells is found in actin-filament-attached plasma membrane regions that are brought together upon TCR patching.

### The distribution of the TCR and its colocalisation with actin filaments is cell cycle dependent

**Figure 3:**
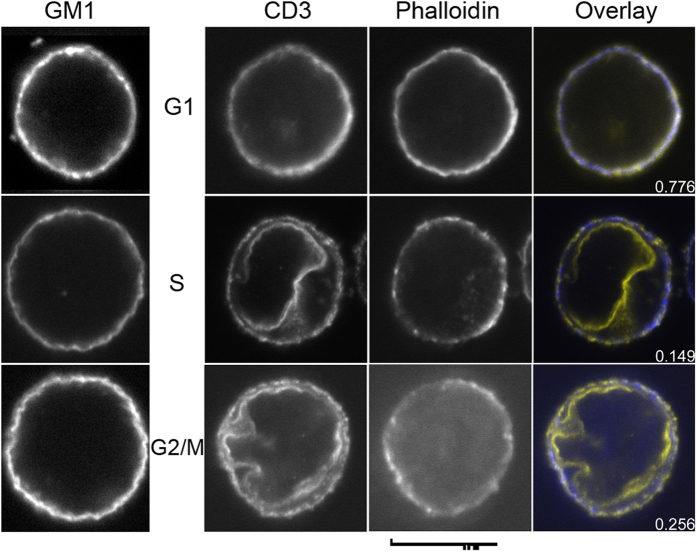
The distribution of the TCR during progression through the cell cycle. Jurkat T cells in different cell cycle phases were fixed and stained with either cholera toxin B subunit Alexa Fluor-594 or the combination anti-CD3(UCHT1)/anti-mouse Alexa Fluor-594 and FITC-phalloidin. The overlay image displays colocalization in white, Alexa Fluor-594 in yellow and FITC in blue. The images displayed are representative of the respective populations of sorted cells. Scale bar 10 μM.

Since actin filaments were enriched under TCR-patches in activated T cells, we next addressed whether the TCR in unstimulated cells was colocalised with actin filaments at the plasma membrane. To this end we studied the correlation between CD3 and actin filaments at the plasma membrane using the Pearson correlation coefficient (PCC) and replicate based noise corrected correlation (RBNCC) in Jurkat T cells. The results were inconclusive with both the CD3 distribution and the PCC varying substantially between cells. We suspected that this large variation was related to distribution changes as the cells were going through the cell cycle and addressed this by synchronising the cells using a double aphidicolin block after which the cells were FACS-sorted to obtain populations of high purity. This indeed revealed some striking cell cycle stage depended distribution differences. In G1 phase, there was intense CD3ε staining of the entire plasma membrane where the PCC between Alexa Fluor-594 and FITC-phalloidin was 0.663±0.048 (Table 2), indicative of a strong colocalisation between actin filaments attached to the plasma membrane and the TCR. However, this high correlation was not seen in cells in S and G2/M phase, where the PCC between CD3ε and actin filaments at the plasma membrane dropped to less than a third of the value seen in G1 phase (Table 2). Interestingly, this drop coincided with a redistribution of a substantial fraction of CD3-staining from the plasma membrane to the perinuclear membrane (Fig. 3). Perinuclear staining was never observed in G1 phase cells, although some cytoplasmic, both diffuse and punctate, CD3ε staining was seen at all cell cycle phases. For comparison we also followed the distribution of the ganglioside GM1 that is exploited as a receptor by cholera toxin^24^ and found that it remained localised at the plasma membrane at all cell cycle phases (Fig. 3).

## Discussion

It is debated whether the TCR constitutively resides in lipid rafts or translocates to lipid rafts upon TCR engagement and although this matter has not been resolved, studies on this topic have petered out and most recent studies on T cell signalling completely ignore the importance of membrane nanodomains with different lipid packing in the signalling process. However, their involvement in T cell signalling is still considered in review articles on signalling models^25^,^26^ and we set out to answer this outstanding question.

It has been claimed that the TCR is transferred to lipid rafts upon engagement and subsequent phosphorylation of its ITAMs^27^,^28^, but these data were based on detergent-extraction methods that can be misleading, for instance due to the risk of membrane rearrangement^29^,^30^. Evidence indicative of the TCR residing in lo-domains prior to its engagement exist^3^,^6^ and our data corroborates the notion that the TCR partitions to lo-domains both before and after its engagement. We found that the lo-domains were enriched in the TCR-patches although the proportion of lo-domains in the plasma membrane was not altered upon TCR aggregation. The most likely explanation is that the TCR always resides in lo-domains. If the TCR instead was translocated to the lo-domains upon patching there must have been a mechanism that would simultaneously translocate the lo-domains to the patches which seems unlikely given that only anti-CD3 and a crosslinking secondary antibody were added to the cells, i.e. the lo-domains must have arrived at the patches with the TCR. We cannot provide an estimate on how many TCRs there are per lo-domain either before or after patching, but our conclusion offers an explanation to the recent finding that the density of TCRs in microclusters is not altered by the potency of the ligands^14^, which you would expect it to be if the TCR was recruited from one type of lipid environment to another. It is not possible to determine the distribution of the TCR between ld- and lo-domains from the GP-values but the lo-domains were enriched in the TCR patches and minimal TCR staining was observed between the patches which is compatible with TCR being found in lo-domains. However, the possibility that ld-domains are trapped in the TCR patches, as they are in newly formed CT-B patches^22^, cannot be excluded.

In our study we have used TCR crosslinking to study the TCR location in relation to plasma membrane lo-domains. TCR crosslinking elicits a signalling response characterised by tyrosine phosphorylation, calcium fluxing, ERK activation and gene expression as does stimulation with antigen-MHC complexes^31^. The main difference between these two means of activating the TCR is that affinities differ by a factor of 10^4^ with the antibodies binding more strongly^32^,^33^. Given that we could not observe any change in the abundance of plasma membrane lo-domains upon either primary or secondary antibody binding, it seems unlikely that this stronger affinity influenced the distribution of the TCR.

Patching of the lipid raft markers GM1 via cholera toxin B and the GPI-anchored protein CD59 leads to T cell activation^3^,^4^. We have recently shown that this patching leads to actin polymerisation at the cell periphery and the formation of lo-domains^22^, which is different from TCR-patching upon which no lo-domains were formed and no actin polymerisation in the cell periphery took place. Still T cell signalling induced by CD59 stimulation is dependent on TCR surface expression^34^ suggesting that the TCR leaves the lo-nanodomains where it normally resides and enters the larger newly formed lo-domains where signalling can proceed. The large lo-domains likely contribute to generating an environment where phosphorylation of signalling molecules, starting with the ITAMs of the TCR/CD3 subunits, can occur in the absence of deactivating phosphatases and in conjunction with other mechanisms like kinetic segregation^35^. That the TCR in resting cells resides in nanodomains increases the chance that the cellular signalling threshold is overcome by allowing serial triggering in the presence of low (as low as one) numbers of agonist pMHC required to elicit a signalling response^36^,^37^.

Many signalling molecules colocalise in microclusters, mainly observed using TIRF microscopy, during early stages of T cell activation^38^,^39^,^40^,^41^. The relationship between these microclusters and lo-domains has not been addressed by assessing the microcluster membrane order, but it has been suggested that the TCR microclusters form independently of lipid raft aggregation based on the FRET finding that several lipid raft markers do not accumulate in the microclusters^42^. However, in order for the FRET signal to increase upon domain coalescence, the density of at least one of the molecules would have to be increased within the aggregated domain and the assumption that this should occur upon domain coalescence remains speculative.

**Figure 4:**
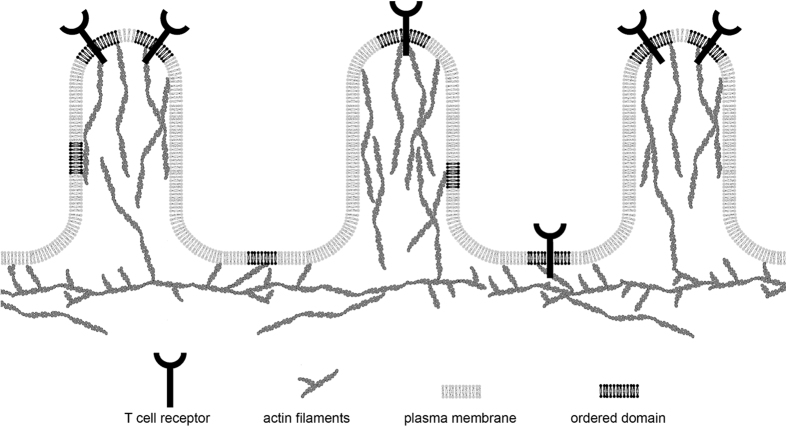
Model of TCR location in resting T cells. The presence of the TCR in lo-domains at the tip of membrane protrusions of actin filaments positions the receptor at an ideal position for scanning the environment. Anchoring of the TCR to the actin filaments may increase its residence time at the cell tentacles.

Cells are not flat^43^ and membrane topography is likely to play a pivotal role in the compartmentalisation of signalling events^44^. For T cell signalling it has, based on the distribution of more than 60 components in the IS, been proposed that signal amplification takes place in the more ruffled membrane areas at the periphery of the IS whereas signalling initiation preferentially occurs in the central area^18^. T cell signalling emanates from small clusters in touch with the antigen presenting cell or a surface coated with stimulatory molecules both of which are consistent with the TCR being found in lo-domains at the tip of membrane protrusions which we propose is the more likely scenario (Fig. 4). This is supported by the cell peripheral actin filaments being more dense in the TCR-patches than at other plasma membrane locations and the previously found correlation between actin filaments and lo domains^22^. From a functional point of view this would be the ideal position for a receptor whose role it is to detect foreign molecules on other cells and it has been established that a large fraction of TCRζ in resting T cells is bound to actin filaments^45^. This does not mean that this is the only location of the TCR since protrusions form and retract continuously and it does not exclude the possibility that potentially ld-phase located TCRs are engaged when an immunological synapse is formed. In this context, it is important to keep in mind that clusters are indistinguishable from the distance of an undulating plasma membrane to the coverslip when TIRF-microscopy is used^44^.

Our finding that the CD3ε to a large extent redistributes from the plasma membrane to the perinuclear membrane of Jurkat T cells in S and G2/M-phase was somewhat puzzling, but from a cell perspective it makes sense that a higher proportion of the TCR is at the cell surface when the cells are in G1-phase (or Go for quiescent cells) and ready to commit to proliferation. An alternative to redistribution is that the perinuclear TCR is newly synthesised and gets delivered to the plasma membrane upon G1-phase entry and that plasma membrane TCR becomes degraded when the cells exit the G1-phase. Perinuclear anti-CD3ε staining is something we have observed earlier^6^ and Jurkat T cells with undetectable CD3 surface expression have recently been reported^34^. Moreover, both TCRα and CD3ε have been reported to localise to the perinuclear membrane in addition to intracellular vesicles and the cell surface^46^,^47^ even if none of these studies used synchronised cells. Together the data suggest that not only CD3ε, but the entire TCR-CD3 complex, relocates to the perinuclear membrane during cell cycle progression to the S-phase and that signalling predominantly occurs during the G1-phase. That no distribution change was observed with GM1 that is both a plasma membrane lipid constituent and a cell surface receptor demonstrates that there is no general plasma membrane redistribution as T cells progress through the cell cycle.

We found that the distribution of the TCR is correlated with actin filaments at the plasma membrane in resting cells and that the aggregated TCR domains in activated T cells were enriched in actin filaments. However, at the cell periphery there was no increase in the extent of polymerised actin upon T cell activation even if rearrangement of the actin cytoskeleton is a hallmark of T cell signalling. The explanation for this probably is that we were measuring the actin filaments at the very edge of the cell, not total actin filaments, and actin has been found to accumulate at a depth up to 2 μm under the IS^18^. Moreover, our results are in line with the recent finding that actin filaments close to the TCR are stable^48^. What our data suggests is that there is no big difference in the fraction of actin filament anchored TCRs between resting and activated T cells since lo-domains form as a consequence of actin filament attachment to the plasma membrane via phosphoinositides^22^ and no change in the plasma membrane lo-domain proportion was observed upon activation. It is also possible that the attachment of plasma membrane protein components, in this case the TCR, to actin filaments can trigger the formation of ordered membrane domains because it creates pinning points^49^.

A recent study highlights the importance of links between actin filaments and the plasma membrane when the distribution of molecules is studied. By assessing the location of the TCR in giant plasma membrane vesicles (GPMVs) and studying the TCR distribution in plasma membrane spheres (PMSs) attached to dead cells it was concluded that the TCR partitions to ld domains^50^. However, although GPMVs are excellent for obtaining a pure plasma membrane fraction and both GPMVs and PMSs have been used to demonstrate that the lipids of the plasma membrane can separate into two co-existing liquid phases^51^,^52^,^53^,^54^, they are less suitable for studies of molecular distributions since they for instance are devoid of their natural links to actin filaments^55^. Consequently, proteins that like the TCR are linked to actin filaments are likely to display differential phase preference when those links are missing especially since lo-domains form where actin filaments are linked to the plasma membrane^22^.

## Methods

### Cell culture

Jurkat T cells (clone E6.1) were from ATCC and cultured as described previously^5^.

### Materials

6-dodecanoyl-2-dimethyl-aminonaphthalene (laurdan), cholera toxin B subunit Alexa Fluor-594 and anti-mouse Alexa Fluor-594 and 647 were from Invitrogen (Carlsbad, CA). Catalase, FITC-phalloidin, glucose oxidase, HEPES, mineral oil and TESPA (3-aminopropyltriethoxy silane) were from Sigma (St Louis, MO). Anti-CD3e clone UCHT1 was a kind gift from Steve Ley, NIMR, UK.

### Isolation of human T lymphocytes

Peripheral blood mononuclear cells were isolated from fresh blood from healthy donors on a Histopaque gradient, mixed with pan T cell isolation beads (Miltenyi Biotec Inc., Auburn, CA). CD3 positive cells were negatively selected on a MACS column. The purified cells were kept in RPMI in humidified incubator and used within two hours.

### Ethical statement

This study was conducted in accordance with the principles expressed in the Declaration of Helsinki and was approved by the Ethical Review Board first in Stockholm (2007/823-31/2) and later in Uppsala (2011/850-32). Informed consent was obtained from all blood donors and venous blood was collected by qualified personnel.

### Cell synchronisation and FACS sorting of synchronised cells

Cells were incubated with 3 μM aphidicolin for 16 h followed by a drug-free interval of 9 h. A second aphidicolin block was administered for another 16 h. Cells were harvested 0.5 h, 3.5 h and 6.5 h after the release of the second block. High purity populations of cells in G1, S and G2/M phase were obtained by cell sorting in a FACSVantage SE (BD Biosciences, Franklin Lakes, NJ) after staining with Hoechst 33342. Sorted cells were stored on ice until further use.

### Cell staining and quantitative analysis of colocalisation

Cells were attached to coverslips, fixed and blocked as described earlier^6^. Cells were stained with either FITC-Phalloidin (0.8 μg/ml) and anti-CD3 (UCHT1, 8 μg/ml) or cholera toxin B subunit Alexa Fluor-594 (2.5 μg/ml) in 2% BSA/PBS for 30 min at RT. Images were acquired with a LSM 510 Meta confocal microscope (Carl Zeiss MicroImaging GmbH, Göttingen, Germany) with a 100x oil objective (NA 1.4). FITC and Alexa Fluor-594 were excited by 488 nm and 543 nm lasers and emission collected between 500–530 nm and above 585 nm respectively. Cells selected for imaging had discernible nuclei and no immediate neighbours. Colocalisation was measured using the Pearson correlation coefficient (PCC) and replicate based noise corrected correlation (RBNCC) where two images from each channel enables the calculation of a correction factor that is used to eliminate the contribution of image noise to the analysis^56^. Imaging and image analysis was performed blind with the operator unaware of which population was being studied.

### Cell staining and live cell imaging

After two washes, cells were suspended at 1.25 × 10^6^ cells/ml and labelled with 5 μM laurdan for 30 min at 37 °C. The cells were then suspended in serum-free HEPES-buffered RPMI medium. 2.5 × 10^5^ cells in 200 μl medium were added to TESPA-coated coverlips, of thickness No 1½, attached to the bottom of Petri dishes. CD3 crosslinking was performed with 8 μg/ml anti-CD3 for 30 min on ice followed by 10 μg/ml anti-mouse Alexa Fluor-647 for 30 min on ice followed by 15 min at 37 °C^6^. The control cells were subjected to the same incubation times at the same temperatures. For assessing whether the temperature changes affected the GP-values, one population of Jurkat T cells was kept continuously at 37 °C whereas another was incubated for 30 min on ice before it was allowed to recover for 15 min at 37 °C. A thin layer of mineral oil floating on cell medium was used to minimise evaporation. Live cell imaging was performed on a wide-field fluorescence Zeiss Axiovert 200 M microscope (Carl Zeiss MicroImaging GmbH, Göttingen, Germany) equipped with a Cascade 1 K camera (Photometrics, Tucson, AZ) in HEPES-buffered RPMI medium. 7000U of catalase and 16U of glucose oxidase were included in the medium to minimise photobleaching^57^,^58^. A 63x water objective lens (NA 1.3) and DG4 (Sutter Instrument, Novato, CA) with 350/50x and HQ577/20x excitation filters, a z365/577rpc dichroic and a dual-view dichroic ms-470LDX (Chroma, Rockingham, VT) and 425/40 m and 51018 m emission filters were used for laurdan and Alexa Fluor-647 imaging. Focus was adjusted under transmitted light and images were acquired avoiding prior exposure to UV or intense excitation light to minimise photobleaching. Z-stacks of eleven images with 200 nm spacing were acquired around the equatorial plane of the cells.

### Estimation of filamentous actin at the plasma membrane

CD3 was crosslinked as above and cells were fixed as described earlier^22^,^59^. Cells were then stained with 0.8 μg/ml FITC-phalloidin for 30 min at RT and 5 μg/ml DiI-C12 for 15 min at RT. Cells were washed three times in PBS and mounted in AF1 (Citifluor Ltd, London UK). Images were acquired using an UltraView ERS spinning disc confocal system (Perkin Elmer, Waltham, MA) connected to an Axiovert 200 M microscope (Carl Zeiss MicroImaging GmbH, Göttingen, Germany). A 63x oil objective (NA 1.4) and an ERS 3E dichroic were used. Excitation of FITC, DiI-C12 and Alexa Fluor 647 was performed with 488 nm, 561 nm and 640 nm laser lines respectively and the UltraView ERS standard emission filters for the three fluorophores. To avoid bleaching affecting the image analysis, focus was adjusted under transmitted light and FITC-phalloidin images immediately acquired once the excitation source was turned on. Points demarcating the plasma membrane were optimised by automatically searching over a short distance around the initial position for the most intense pixel along a line originating at the centre of the cell. Once delineated, the mean fluorescence intensity per pixel of the corresponding image of FITC-phalloidin was calculated. The membrane marker DiI-C12 was used to define the plasma membrane as a region of interest.

### Ratiometric analysis

Image stacks from both laurdan channels were deconvolved together with the Alexa Fluor-647 conjugate image stack of a plasma membrane molecule using AutoQuantX (Media Cybernetics, Bethesda, MD). The deconvolved images were checked for alignment using a cross correlation function and the plasma membrane was demarcated as described above. A single line of pixels, between sequential points, was used to select pixels corresponding to the plasma membrane. The background intensity, based on an area outside the cell, was subtracted. The calculation of the ratio between the two laurdan channels used the generalised polarisation formula:
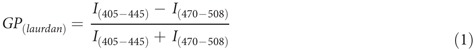


The average ratio over the whole plasma membrane was obtained from the arithmetic mean of the ratios for individual pixels. Fluorescent staining of CD3 was used to select patched and non-patched areas of the plasma membrane. The unitary scale bar was used to display distances^59^. All in house software used a Semper6w kernel (Synoptics Ltd, Cambridge, UK). Figures were prepared using Adobe Photoshop 7.0 software.

### Statistical analysis

A two-tailed t-test was used to compare populations and a paired two-tailed t-test was used to compare different regions of the same cells.

## Additional Information

**How to cite this article**: Dinic, J. *et al*. The T cell receptor resides in ordered plasma membrane nanodomains that aggregate upon patching of the receptor. *Sci. Rep.* **5**, 10082; doi: 10.1038/srep10082 (2015).

## Supplementary Material

Supplementary Information

## Figures and Tables

**Table 1:** Actin filaments are concentrated under patches of CD3 in the plasma membrane.

**Experimental group**	**FITC-phalloidin intensity in pm pixels (relative values)**	**N**
Control, whole pm	11.7±0.56	43
CD3, whole pm	13.0±0.60	41
CD3, non-patched regions	12.3±0.57***	41
CD3, patched regions	18.4±1.3***	41

Jurkat T cells were crosslinked for CD3, fixed and stained with FITC-phalloidin. pm=plasma membrane. Data shown are means ± s.e.m.

^***^p<0.001 for a paired two-tailed t-test with the whole pm of CD3-patched cells.

**Table 2:** Colocalisation between CD3 and actin filaments at the plasma membranes of Jurkat T cells.

**Cell cycle phase**	**Pearson correlation coefficient**	**p-value**	**N**
G1	0.663±0.048		7
S	0.209±0.067	6.5×10^−4^	5
G2/M	0.203±0.050	2.5×10^−5^	7

Data shown are means ± s.e.m. p values are from a two-tailed t-test with the G1 phase population.
