# Supplementary material for: The T cell receptor resides in ordered plasma membrane nanodomains that aggregate upon patching of the receptor
Source: Sci Rep. 2015 May 8;5:10082. doi: 10.1038/srep10082 (PMC5386217; doi:10.1038/srep10082)
Supplement: Supplementary Information [file srep10082-s1.pdf]

Supplementary Information.

The T cell receptor resides in ordered plasma membrane nanodomains that aggregate upon patching of the receptor

Jelena Dinic, Astrid Riehl, Jeremy Adler and Ingela Parmryd

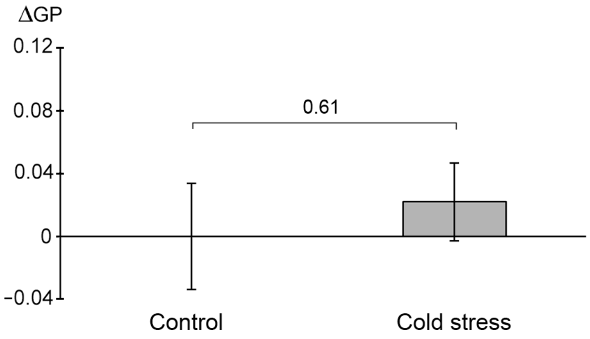

Figure S1. The relative proportion of lo and ld domains in the plasma membrane of Jurkat T cells does not reflect previous temperature changes. Laurdan-labelled cells were imaged at 37°C after either having been incubated on ice for 30 min followed by recovery at 37°C for 15 min or being kept at 37°C throughout the experiment. The GP values for the control cells were normalized to 0. Data shown are mean ± s. e. m.. p values are from a two-tailed t-test with the control cells.
